# Predictors of mental distress among substance abusers receiving inpatient treatment

**DOI:** 10.1186/1747-597X-5-15

**Published:** 2010-07-07

**Authors:** Ellen Hoxmark, Mary Nivison, Rolf Wynn

**Affiliations:** 1 Department of Substance Use and Specialized Psychiatric Services, University Hospital of Northern Norway, 9291 Tromsø, Norway; 2 Institute of Clinical Medicine, University of Tromsø, 9037 Tromsø, Norway; 3 Psychiatric Research Centre of Northern Norway, University Hospital of Northern Norway, 9291 Tromsø, Norway

## Abstract

**Background:**

Mental distress measured by the HSCL-10 is used as an indicator of psychiatric disorders in population studies, where a higher level of mental distress has been shown to be related to demographic factors such as living conditions and level of education. The first aim of the study was to explore whether mental distress could be a valuable concept in substance use treatment. The second aim of the study was to explore to what degree mental distress among substance users at admission to treatment could be explained by the same demographic factors as in population studies, or whether treatment differences or differences in substance use would be better predictors of mental distress in this population.

**Methods:**

Patients (N = 185) who received inpatient substance use treatment in five different settings in Northern Norway participated in the study. HSCL-10 was used as a measure for mental distress at admission to treatment. The self-report measures AUDIT, DUDIT and DUDIT-E were used for measuring substance use and readiness for treatment. The patients' clinicians reported demographic and treatment factors. A three-block hierarchical multiple regression analysis was conducted to determine potential predictors of mental distress. Block 1 included demographic variables, Block 2 included treatment variables, and Block 3 substance use variables.

**Results:**

Patients generally reported a high level of mental distress at admission to treatment, and 83% reported mental distress higher than the established cut-off level. Being female, having previously received psychiatric treatment, having a higher score on DUDIT and AUDIT, and using a larger number of substances all predicted a higher level of mental distress. The model explained 32% of the variance in mental distress.

**Conclusions:**

Mental distress measured by the HSCL-10 can be a valuable concept in substance use treatment. The HSCL-10 can be useful in screening for patients who are in need of further assessment for psychiatric disorders. Female gender, previous psychiatric treatment, and higher use of substances all predicted a higher level of mental distress. The study underlines the importance of assessing the mental health of patients in substance use treatment.

## Introduction

Clinical and epidemiological studies indicate high rates of co-occurring psychiatric disorders among people with substance use disorders (SUD) [1-6]. Psychiatric comorbidity is the co-occurrence of one or more psychiatric disorders during a period of time [7]. Comorbidity between anxiety disorders, depressive disorders, and SUD is particularly common [8]. In population studies, co-occurring psychiatric disorders are seen in 30-40% of people with alcohol disorder and 40-50% of those abusing other substances [1-3,9-11]. The incidence of psychiatric disorders among individuals with SUD in treatment is even higher [6]. In a prior study, Landheim et al. [12] studied a treatment-seeking sample of patients with SUD in two counties in the southern part of Norway, and found a prevalence of any Axis 1 disorder of 85%; thereof 60% for affective disorders and 78% for anxiety disorders.

Psychiatric disorders have in several studies been found to influence the long-term course of SUD [13-15], and treatment of these disorders affects outcomes of substance use treatment [16]. In a follow-up six years after treatment, Landheim et al. [17] found that major depression together with early onset of SUD were independent predictors of relapse. In another recent longitudinal study of patients with heroin dependence, major depression was the diagnosis most consistently associated with poor outcomes after three years [18].

Given the high rates of co-occurring disorders, and the impact these disorders have on treatment, it is crucial to detect who is in need of further assessment and treatment for psychiatric disorders as soon as possible after admission to substance use treatment. Patients entering substance use treatment should routinely be screened for comorbidity [19].

The screening of psychiatric disorders among substance abusers is nevertheless problematic because of the symptom overlap of substance-induced symptoms and symptoms of depression and anxiety. The relationship between anxiety symptoms, affective symptoms and substance-induced symptoms is complex. Substances can be used as self-medication and thereby conceal symptoms of anxiety and depression, or symptoms can be a result of substance abuse [20]. An example of the latter is that symptoms of anxiety and depression among alcohol dependent patients at admission to treatment often have been shown to spontaneously recede during treatment [21,22].

Mental distress can be used as a measure for detecting individuals in the general population who are in need of further examination or psychiatric treatment. The Hopkins Symptoms Checklist-10 (HSCL-10) [23] is a screening instrument for detecting mental distress, commonly used in population studies. The HSCL-10 is based on the original HSCL-90 version, using two out of originally nine dimensions [24,25]. In a Norwegian population study, the established cut-off for HSCL-10 yielded a prevalence rate of 11.4% [23]. The literature suggests that 50 - 60% of the "cases" identified by instruments like HSCL-10 most likely will qualify for one or more psychiatric diagnoses following a clinical assessment [26].

The level of mental distress has been found to be higher among women, among people with little education, and among the unmarried and those living alone [27-31]. A higher level of mental distress has also been shown to be related to economic problems [32], poor social support [33] and belonging to an ethnic minority [34]. Typically, those in lower socio-economic groups have worse health and higher mortality than those in higher socio-economic groups [35]. SUD and other psychiatric disorders are generally associated with a variety of psychosocial risk factors [36-38]. The National Comorbidity Survey (1990 - 1992) demonstrated that not having an occupation was the demographic factor with the strongest association with SUD [39].

### Aims of the study

In this study, we measured mental distress one week prior to admission to treatment. The first goal of the study was to detect the level of mental distress among substance abusers seeking inpatient treatment, and thereby examine whether mental distress at admission to treatment could be a valuable concept in substance use treatment. The second goal of this study was to explore what impact demographic variables, treatment variables, and substance use variables had on mental distress.

## Methods

### Treatment settings and participants

Treatment for SUD in Northern Norway is given primarily in inpatient settings, e.g. only 2% of the referrals to substance use treatment in the area during the first half of 2005 were referrals to out-patient clinics [40]. The present study was conducted in five public inpatient units for treatment of SUD in Northern Norway. These constituted the inpatient treatment offered for patients with SUD within the largest hospital in Northern Norway. The units serviced primarily patients in the Districts of Nordland, Troms, and Finnmark (500 000 inhabitants), and included approximately 60% of the available treatment beds for SUD patients in the whole region. One of the units was a therapeutic community, one a short-term/acute (up to six weeks) unit, one unit specialized in the assessment and treatment of dual diagnosis, and two were long-term (six months) units. All units treated men and women and patients with all types of SUD. In the units, treatment was given in both group and individual settings. Patients were treated with a combination of network-based approaches, psychological therapies and pharmacological treatment, although the different elements were given different emphasis in the various units. The number and proportion of participants from each unit is shown in Table 1. A large proportion of the patients had been treated in other units previously. Each unit was small, and it was necessary to pool data from different units to achieve a substantial number of subjects, and to cover a wider range of patients.

**Table 1:** Participation in the different units

Unit	Admitted in the period of inclusion	Participating in study	The proportion of participators from each unit (%)	Mean age (SD)
Dual diagnosis ward	43	13	30	29.15 (7.7)
Therapeutic community	37	26	70	27.50 (6.60)
Short term unit	122	58	48	37.72 (11.78)
Long term unit 1	53	42	79	41.26 (9.27)
Long term unit 2	75	46	61	41.83 (10.24)

Total	330	185	56	37.51 (11.16)

Patients were recruited on entry to the units. In the study period, 330 patients were admitted to the units. Patients who were considered not able to give an informed consent (N = 18) or whose hospital stay was too short to be included (N = 31) were not asked to participate. In all 201 patients agreed to participate and signed an informed consent form. Subjects (N = 16) with three or more missing items in HSCL-10 were excluded from the analyses. The final sample thus consisted of 185 respondents. This gave a participation rate of 56%. The majority of the sample were men (71%) and the average age in the sample was 38 (SD = 11.2; range 18 - 63 years). 97% of the sample was born in Norway, 96% of their mothers and fathers were also born in Norway. Our sample, compared with the whole sample of SUD patients seeking treatment in Northern Norway in the same period of time, did not differ significantly with regard to gender, age, education, occupation, living condition, marital status, current medical treatment, previous substance use or psychiatric treatment, or admittance under legal circumstances.

### Measures

#### Dependent variable: mental distress

Symptoms of mental distress occurring the last week before admission were measured using the HSCL-10 [23]. The original Hopkins Symptom Check List (HSCL-90) was a self-rating questionnaire that assessed psychopathology and mental distress, and consisted of 90 items that formed nine symptom dimensions or scales [24,25]. The HSCL-90-R has proven to be an adequate screening instrument for comorbid psychopathology among patients with SUD [41-45]. The HSCL-10 is a shortened version of the HSCL-90-R consisting of two (anxiety and depression) of the original nine symptom dimensions [23]. The HSCL-10 has shown good psychometric properties, has performed almost as well as the full HSCL-90 version, and has shown good qualities in detecting mental distress in the general public [23,46].

The HSCL-10 consists of 10 items on a four-point scale, ranging from 1 (not at all) to 4 (extremely). The participants reported on the following questions: Have you in the course of the past week been troubled by feeling: suddenly scared for no reason, fearful, faint or dizzy, tense or keyed up, guilty, difficulties falling asleep, blue, worthlessness, that everything is an effort, hopeless about the future. The average item score (General Severity Index) was calculated by dividing the total score with the number of items answered, thus the score ranged between 1.00 and 4.00. When one or two items were missing we divided the individual total sum with the number of the items the individual had answered (i.e. eight or nine). An average score of 1.85 or above has shown to be a valid predictor of mental distress in the normal population [23]. The scale was used as a continuous variable in our study.

### Predictors

Use of substances was measured by the Alcohol Use Disorders Identification Test (AUDIT) [47] and the Drug Use Disorders Identification Test (DUDIT) [48]. Both are screening instruments for identifying persons with SUD. Both instruments are standardized and based on selected criteria for substance abuse, harmful use and dependency, according to the ICD-10 and DSM-IV diagnostic systems. Treatment motivation and current use of specific substances were measured by the self-report Drug Use Disorders Identification Test - Extended (DUDIT-E) [49]. A Motivational Index was formed from three factors in DUDIT-E (positive and negative aspects relating to substance use, and treatment readiness). Use of specific substances was dichotomized between using the substance twice a week or more often, and using the substance less often [50]. The number of substances was measured by adding up the number of drugs used twice a month or more often (as reported on DUDIT-E) in addition to alcohol used twice a week or more often (as reported on AUDIT).

Demographic information and the patients' treatment histories were assessed using the Norwegian National Client Assessment Form [51]. Variables in this form include age, sex, occupation, housing, and previous treatment. This form is routinely completed for all patients admitted to substance use treatment in Norway.

### Procedures

From September 2007 to December 2008, all patients being treated in the units, and who were considered able to give an informed consent, were given written and oral information about the study, and invited to participate in the study by one of the research collaborators who worked in the units. The participating patients completed a questionnaire as soon after admittance as possible, depending on withdrawal symptoms or other symptoms. The questionnaire asked about conditions prior to admittance. It included questions about substances used (AUDIT, DUDIT, DUDIT-E), mental distress (HSCL-10) and motivation for treatment (DUDIT-E). As compensation for filling out the questionnaire, the participants could choose between one cinema ticket or two lottery tickets. Through the informed consent procedure, the participants granted us access to the clinicians' assessments and other routinely collected data. This included information on demographic variables and previous treatment.

### Analyses

All analyses were conducted using the Statistical Package for Social Sciences 16.0 (SPSS 16.0). Reliability was assessed with Cronbach alpha [52]. Descriptive statistics were used to examine the frequency of substance use [53]. Correlational analyses and t-tests were used to identify substance abuse and demographical factors associated with mental distress. A hierarchical multiple regression analysis was conducted with the score on HSCL-10 as the dependent variable, and demographic variables, treatment variables and substance abuse variables as predictors. Regression diagnostics were performed to test for collinearity, outliers and the overall fit of the models.

The study was reviewed and approved by the Regional Committee for Medical Research Ethics (REK Nord) and by the Norwegian Social Science Data Services (NSD).

## Results

### Mental distress and substance abuse

A high proportion of the participants scored above the cut-off point for HSCL-10 (N = 154, 83%). The mean score was 2.58 points (SD = 0.75). The internal consistency of the HSCL-10 was high in the sample (Cronbach's alpha = 0.90). Level of mental distress measured by the HSCL-10 did not differ significantly between the five participating units (Table 2).

**Table 2:** Scores on HSCL-10, AUDIT, DUDIT and DUDIT-E according to unit

Unit	Mean score HSCL-10 (SD)	Mean score AUDIT (SD)	Mean score DUDIT (SD)	Mean score DUDIT-E (SD)	Mean number of substances (SD)
Dual diagnosis ward	2.92 (0.71)	17.31 (9.72)	29.23* (10.16)	6.86 (5.88)	2.92 (1.61)
Therapeutic community	2.75 (0.42)	12.12* (9.73)	35.04** (6.76)	7.07 (4.51)	4.12** (1.99)
Short term unit	2.71 (0.69)	12.72** (12.60)	24.82* (14.20)	4.22 (3.23)	2.93 (1.57)
Long term unit 1	2.36 (0.86)	23.17** (8.98)	12.08** (13.93)	3.93 (4.79)	2.07* (1.76)
Long term unit 2	2.42 (0.78)	21.58* (10.98)	14.33** (15.34)	5.15 (8.82)	2.09** (1.93)

Total	2.58 (0.75)	17.51* (11.77)	21.14 (15.57)	5.13 (5.73)	2.69 (1.88)

*p < 0.05; **p < 0.01.

Alcohol (61%) and hypnotics (60%) were the most common substances, followed by cannabis (46%), analgesics (46%), amphetamine (35%) and opiates (33%). Only a small proportion of the sample reported using cocaine (7%), hallucinogens (4%), GHB and other substances (3%) or solvents (0.5%). A high proportion of the sample consisted of poly-substance abusers, with 74% using two or more substances and 58% using three or more substances twice a week or more often. There were significant differences in the severity of substance abuse between the participating units. Patients in the therapeutic community reported a higher score on DUDIT than the rest of the sample, a higher number of substances used, and a lower score on AUDIT. The patients in the short term unit reported a lower score on AUDIT and a higher score on DUDIT than the rest of the sample whereas patients in the dual diagnosis ward reported a higher score on DUDIT than the rest of the sample. The patients in the two long term units reported a higher score on AUDIT, a lower score on DUDIT and a lower number of substances used than the rest of the sample.

### Predictors of mental distress

As a preliminary step in the statistical analysis, we performed univariate correlational analyses and t-tests between individual HSCL-10 scale scores and three types of variables: demographic variables (age, gender, marital status, living conditions, employment, and education), treatment variables (motivational index, previous substance use and psychiatric treatment, prescribed medication for psychiatric problems last four weeks before admission, and legal circumstances at admission) and severity of substance abuse (score on AUDIT, DUDIT and number of substances used) (Table 3). We found significant correlations and t-test differences between the HSCL-10 scores and previous treatment for psychiatric problems, prescribed medication for psychiatric problems last four weeks before admission, score on DUDIT, number of substances used, gender, and employment. An interaction effect between previous treatment for psychiatric problems and prescribed medication for psychiatric problems last four weeks was resolved by including an interaction variable in the subsequent regression analysis.

**Table 3:** HSCL-scores in relation to demographic characteristics, current substance use, patterns of substance use, and treatment history for 185 substance abusers (univariate analyses)

N = 185	M or n	Range, % or SD	t	r	Sig
**Demographic characteristics**					
Mean age (years)	38	(18 - 63)		-.13	0.069
Male	131	(71%)	3.08		**0.002**
Gainfully employed	40	(22%)	1.97		**0.050**
Single, never married	156	(84%)	0.38		0.707
Living alone	121	(65%)	1.70		0.083
Post-school qualification	99	(54%)	0.004		0.997
**Severity and pattern of substance use**					
AUDIT	17.51	(11.77)		0.06	0.426
DUDIT	21.14	(15.57)		0.36	**0.001**
Number of substances used	2.7	(1.9)		0.36	**0.001**
**Treatment history**					
Undergoing medical treatment for psychiatric problems	92	(50%)	3.57		**0.001**
Previous treatment for psychiatric problems	126	(68%)	3.82		**0.001**
Previous substance abuse treatment	154	(83%)	1.10		0.273
Admitted under legal circumstances	4	(2%)	1.25		0.213
Motivation for treatment (DUDIT-E) (N = 146)	5.13	(5.73)		0.13	0.122

We then performed a hierarchical multiple regression analysis with the demographic variables and the significant treatment variables together with variables concerning severity of substance abuse as predictors and HSCL-10 as the dependent variable (Table 4). The demographic variables age, sex, never married, living conditions, employment and education were entered into the first block and accounted for 8% of the variance in HSCL-10 scores [F(6,178) = 2.63]. The second block included previous treatment for psychiatric problems, prescribed medication for psychiatric problems the past four weeks and interaction between the two variables, and explained an additional 9% of the variance [F(9,175) = 4.08]. Block three added measures of severity of substance abuse (DUDIT, AUDIT and number of substances) and explained an additional 15% of the variance [F(12,172) = 6.72].

**Table 4:** Determinants of HSCL-10--scores: contributions of each variable block to changes in *R*^2^

Determinant	***R***^**2**^	**Δ*R***^**2**^	*F*	*df*	Δ*F*	Sig Δ*F*
Block 1: Demographics	0.081	0.081	2.628	6,178	2.628	**0.018**
Block 2: Previous psychiatric treatment	0.174	0.092	6.507	9,175	4.083	**0.001**
Block 3: Substance abuse severity	0.319	0.146	12.258	12,172	6.718	**0.001**

A hierarchical multiple regression strategy was used in which blocks of variables were added to the regression equation sequentially. *R*^2^, *F *and *df *refer to the overall regression equation after each block has been entered into the model; Δ*R*^2^, Δ*F*, and significance Δ*F *describe the contributions of each individual block.

Summary statistics for the complete model are presented in Table 5. The final regression model accounted for a significant 32% of the explained variance in HSCL-scores [F(12,172) = 12.26]. Previous psychiatric treatment (p = 0.037), score on AUDIT (p = 0.007) and DUDIT (p = 0.05) and number of substances used (p = 0.013) were significantly related to the score on HSCL-10 in the complete model. Gender (p = 0.014) was the only demographic variable that was a significant predictor in this model. Women, patients who had a previous experience of psychiatric treatment, patients with a higher score on AUDIT and DUDIT, and those using a higher number of substances, reported higher HSCL-10 scores.

**Table 5:** Final stage in the hierarchical multiple regression of mental distress measured by HSCL-10

Determinant	B	SE	Standardized B	*p*
Gender	-0.270	0.109	-0.165	**0.014**
Age	0.003	0.005	0.050	0.525
Gainfully employed	-0.057	0.118	-0.032	0.628
Never married	-0.193	0.139	-0.094	0.167
Living alone	-0.013	0.110	-0.008	0.908
Post school qualifications	-0.081	0.101	-0.055	0.424
Current medical treatment	0.110	0.190	0.074	0.565
Previous psychiatric treatment	0.291	0.139	0.182	**0.037**
AUDIT Sum	0.012	0.005	0.196	**0.007**
DUDIT Sum	0.013	0.005	0.283	**0.005**
Number of substances used	0.086	0.034	0.217	**0.013**

## Discussion

Symptoms of mental distress were highly prevalent among patients with SUD being admitted to inpatient treatment. We found that more than 80% of the patients had a level of mental distress above the established cut-off level of 1.85 on the HSCL-10. The level in the normal population has been estimated to 11.4% [23]. The level of mental distress in our study corresponds to the prevalence of Axis 1 disorders found by Landheim et al. [12], but is higher than is found in substance abusers in population studies [2,9,11]. Previous studies have estimated that around 50 - 60% of the "cases" identified by screening instruments such as HSCL-10 actually qualify for a psychiatric diagnosis. While many of the patients with a score on mental distress above cut-off probably were in need of some type of psychiatric treatment, it is likely that this cut-off level also captures patients who are not in need of specialized psychiatric services [26]. The high rates of mental distress found in this study could reflect the fact that starting treatment is a stressful experience. Further studies should be performed in order to examine which cut-off level on the HSCL-10 that is optimal for clinical use for SUD inpatients.

Being female was the only demographic factor that significantly predicted the HSCL-10 scores. Surveys in the general public generally report a higher level of mental distress for women than for men [23,27,29,30]. Our study had similar results: women reported a higher degree of mental distress according to the HSCL-10 compared to men. It remains an open question as to whether women actually experienced more distress than men, or whether men underreported their distress. The indicators of social situation used in this study are the same as have been shown to influence the level of mental distress in the general population: marital status, education, living conditions, and occupation [23,28]. In the preliminary univariate analyses only occupation was significant, which means that patients gainfully employed or under education reported a significantly lower level of mental distress compared to the unemployed. In the regression analysis, this factor no longer predicted mental distress. Typically, those in lower socio-economic groups have worse health and higher mortality than those in higher socio-economic groups [35]. The reason that socio-economic status does not predict the level of mental distress in this sample may be that substance abusers in treatment generally have a lower socio-economic status than the general population [37,38].

We were interested in how previous and current treatment for psychiatric problems would influence the level of mental distress at admission to treatment, as these factors were believed to be associated with a particularly vulnerable sub-group of patients (i.e. patients that had demonstrated a need for some form of psychiatric treatment). However, after controlling for an interaction effect between previous and current treatment for psychiatric problems, we found that only previous psychiatric treatment predicted a higher level of mental distress, whereas current psychiatric treatment did not. Although we do not have a clear cut explanation for this finding, it may be that patients who are currently in psychiatric treatment in part are protected against especially high levels of mental distress, while having undergone such treatment in the past does not offer the same protection - which is why they have an increased level of mental distress at admission.

The most powerful predictor for mental distress at admission to treatment was the participants' level of substance abuse. Level of alcohol use as measured by the AUDIT, the level of drug use as measured by the DUDIT and the number of substances used all predicted a higher level of mental distress. A question one may ask is to what degree the level of mental distress results from the use of substances or from stress related to admission to treatment, and whether these symptoms could be reduced during substance abuse treatment. Previous studies have indicated that psychiatric symptoms can be substantially reduced during substance abuse treatment [21,22]. Even though the predictors connected to severity of substance use were the most powerful predictors in our study, these are the predictors that most likely will loose their power during treatment.

The high prevalence of mental distress that was found in this study among substance abusers in treatment emphasizes that it is necessary to screen, assess, and treat psychiatric disorders in substance use treatment facilities. Expertise in assessing and treating these disorders should therefore be available in substance use treatment.

### Strengths and limitations

This is one of few studies in Norway that survey mental distress among substance abusers in treatment, and the first in the northern part of Norway. This part of Norway is a remote area with a particularly high incidence of amphetamine use. Our study covered the range of treatment facilities that is most commonly offered substance abusers in the area. Northern Norway is a relatively well defined catchment area where most of the treatment is given in inpatient settings.

A limitation in the study was the relatively low participation rate. Another limitation was that we assessed mental distress the last week prior to admittance to treatment. It is possible that the level of mental distress before entering treatment was influenced by stress connected to treatment admission, as well as influenced by substances used or withdrawal symptoms.

## Conclusions

Substance abusers seeking inpatient treatment reported on average a high level of mental distress as measured by the HSCL-10. An increased severity in the use of substances, a higher number of substances used, having previously received treatment for psychiatric disorders, and being female all predicted a higher level of mental distress. The findings in this study suggest that mental distress measured by the HSCL-10 could be a valuable concept in substance use treatment by identifying patients who are in need of further examination and treatment for their psychiatric disorders. The study underscores the importance of screening, assessing, and treating psychiatric disorders in SUD patients, and also the importance of having the appropriate expertise available in substance use treatment facilities.

## Competing interests

The authors declare that they have no competing interests.

## Authors' contributions

EH participated in designing the study, collecting the data, analysing and interpreting the data, and in drafting and revising the manuscript. MN participated in designing the study, interpreting the data, and drafting and revising the manuscript. RW participated in designing the study, analysing and interpreting the data, and in drafting and revising the manuscript. All authors read and approved the final manuscript.
